# Supplementary material for: Isolation and Molecular Characterization of Indigenous Penicillium chrysogenum/rubens Strain Portfolio for Penicillin V Production
Source: Microorganisms. 2023 Apr 26;11(5):1132. doi: 10.3390/microorganisms11051132 (PMC10221864; doi:10.3390/microorganisms11051132)
Supplement: Supplementary file 1 [file microorganisms-11-01132-s001.zip › microorganisms-2275507-supplementary.pdf]

Isolation, and molecular characterization of Indigenous *Penicillium chrysogenum/rubens* strain Portfolio for Penicillin V Production

Amol M. Sawant[1,2], Vishwambar D. Navale[1,2], Koteswara Rao Vamkudoth [1,2*]

[1]Biochemical Sciences Division, CSIR-National Chemical Laboratory, Pune-411008, India

[2]Academy of Scientific and Innovative Research (AcSIR), Ghaziabad - 201002, India

*v.koteswara@ncl.res.in

Table S1- Isolation, identification, morphological and molecular identification of fungal *spp*. isolated from food and environmental samples.

**Sample**	**Isolation source**	**Isolate Name**	**Morphological characterization on CYA media plate**				
			**Colony texture**	**Colour aerial**	**Color reverse**	**Degree of growth**	**exudate**
Soil	Andhra Pradesh	BIONCL P1	Velvety	Light green	Pale yellow	Moderate to good	present
Soil	Telangana state	BIONCL P2	Velvety	Light green	Pale yellow	Good	Present
Air	Telangana state	BIONCL P3	Floccose to velvety	Green	Brownish yellow	Moderate to good	present
Air	Telangana state	BIONCL P4	Floccose to velvety	Green	Brownish yellow	Moderate to good	present
Soil	Telangana state	BIONCL P5	Floccose	Creamy white	Pale yellow	Weak to moderate	present
Food	Andhra Pradesh	BIONCL P6	Velutinous	Bluish green	Pale yellow	Moderate	Absent
Soil	*Rajasthan*	BIONCL P7	Velutinous	Creamy white	Yellow	Moderate	Absent
Food	*Gujarat*	BIONCL P8	Floccose	Creamy white	Pale yellow	Weak to moderate	Present
Food	*Rajasthan*	BIONCL P9	Floccose	Creamy white	Yellow	Weak to moderate	Present
Air	*Andhra pradesh*	BIONCL P10	Floccose to velutinous	Light green	Brownish yellow	Moderate to good	Present
Air	*Telangana state*	BIONCL P11	Floccose	Creamy white	Pale yellow	Moderate to good	Present
Soil	*Telangana state*	BIONCL P12	Velvety	Green	Yellow	Good	Absent
Soil	*Andhra pradesh*	BIONCL P13	Floccose to velutinous	Creamy white	Yellow	Moderate to good	Present
Soil	*Maharasht ra*	BIONCL P14	Velutinous	Light green	Yellow	Moderate to good	Present
Soil	*Maharasht ra*	BIONCL P15	Velutinous	Bluish green	Pale yellow	Moderate to good	Absent
Soil	*Rajasthan*	BIONCL P16	Velvety	Light green	Yellow with brown centre	Moderate to good	Absent
Food	*Andhra pradesh*	BIONCL P17	Floccose	Greyish blue	Greyish to yellow	Moderate to good	Present
Food	*Andhra pradesh*	BIONCL P18	Floccose to velutinous	Light green	Pale yellow	Good	Present
Soil	*Andhra pradesh*	BIONCL P19	Velvety	Green	Pale yellow	Moderate to good	Present
Soil	*Andhra pradesh*	BIONCL P20	Floccose to velvety	Light green	Pale yellow	Moderate to good	Present

Food	*Andhra pradesh*	BIONCL P21	Floccose to velutinous	Bluish green	Pale yellow	Weak to moderate	absent
Grain	*Gujarat*	BIONCL P22	Velvety	Creamy white	Yellow	Moderate	Present
Soil	*Maharashtra*	BIONCL P23	Velutinous	Light bluish green	Brownish yellow	Moderate to good	Present
Air	*Gujarat*	BIONCL P24	Velutinous	Creamy white	Yellow	Moderate to good	Present
Soil	*Gujarat*	BIONCL P25	Floccose to velutinous	Dark green	Pale yellow	Moderate to good	Present
Soil	*Gujarat*	BIONCL P26	Floccose	Green	Pale yellow	Moderate to good	Present
Soil	*Gujarat*	BIONCL P27	Floccose	Light green	Brownish yellow	Moderate to good	Absent
Air	*Gujarat*	BIONCL P28	Floccose	Creamy white	Brownish yellow	Weak to good	Present
Food	*Gujarat*	BIONCL P29	Velutinous	Light green	Pale yellow	Moderate to good	Present
Food	*Gujarat*	BIONCL P30	Velutinous	Green	Pale yellow	Moderate to good	Present
Grain	*Andra pradesh*	BIONCL P31	Velvety	Green	Yellow	Moderate	Present
Food	*Gujarat*	BIONCL P32	Velutinous	Light green	Pale yellow	Moderate	Absent
Grain	*Gujarat*	BIONCL P33	Floccose	Creamy white	Brownish yellow	Moderate to good	Present
Soil	*Gujarat*	BIONCL P34	Velvety	Green	Brownish yellow	Good	Present
Soil	*Gujarat*	BIONCL P35	Velvety	Bluish green	Pale yellow	Moderate to good	Absent
Grain	*Andra pradesh*	BIONCL P36	Floccose	Creamy white	Pale yellow	Weak to moderate	Present
Food	*Gujarat*	BIONCL P37	Velutinous	Green	Yellow	Moderate	Absent
Food	*Gujarat*	BIONCL P38	Velutinous	Light green	Pale yellow	Moderate to good	Present
Air	*Gujarat*	BIONCL P39	Floccose	Creamy white	Yellow	Weak to moderate	Present
Air	*Gujarat*	BIONCL P40	Floccose	Creamy white	Pale yellow	Weak to moderate	Present
Food	*Gujarat*	BIONCL P41	Velutinous	Light green	Yellow	Moderate	Present
Food	*Gujarat*	BIONCL P42	Velutinous	Green	Pale yellow	Weak to moderate	Present
Food	*Gujarat*	BIONCL P43	Velvety	Light green	Dark yellow	Moderate	Absent
Soil	*Maharashtra*	BIONCL P44	Floccose to velutinous	White with blackish border	Brownish yellow	Good	absent

Air	*Maharashtra*	BIONCL P45	Velvety	Dark green	Brownish yellow	Good	absent
Soil	*Maharashtra*	BIONCL P46	Velvety	Light green	Brownish yellow	Moderate to good	Present
Soil	*Maharashtra*	BIONCL P47	Floccose to velvety	Dark green	Pale yellow	Moderate to good	Present
Soil	*Maharashtra*	BIONCL P48	Floccose	Bluish green	Brownish yellow	Good	Absent
Soil	*Maharashtra*	BIONCL P49	Velvety	Bluish green	Dark yellow	Good	Present
Soil	*Maharashtra*	BIONCL P50	Velvety	Green	Pale yellow	Moderate to good	Present
Food	*Maharashtra*	BIONCL P51	Floccose to velutinous	Light green	Yellow	Moderate to good	Present
Food	*Maharashtra*	BIONCL P52	Floccose to velutinous	Bluish green	Brownish yellow	Moderate to good	Present
Food	*Maharashtra*	BIONCL P53	Floccose	White	Pale yellow	moderate	Absent
Food	*Maharashtra*	BIONCL P54	Floccose to velutinous	White with black border	Dark yellow	Good	Absent
Grain	*Maharashtra*	BIONCL P55	Floccose	Whitish green	Pale yellow	Moderate	Present
Food	*Maharashtra*	BIONCL P56	Floccose	Whitish green	Pale yellow	Weak to moderate	Absent
Food	*Maharashtra*	BIONCL P57	Floccose to velutinous	Bluish green	Pale yellow	Moderate to good	Present
Soil	*Maharashtra*	BIONCL P58	Floccose	Whitish black	Yellow	Weak to moderate	Absent
Air	*Maharashtra*	BIONCL P59	velutinous	White	Brownish yellow	good	Absent
Soil	*Maharashtra*	BIONCL P60	floccose	Whitish black	Yellow	Weak to moderate	Absent
Soil	*Maharashtra*	BIONCL P61	Floccose	Whitish black	Yellow	Good	Absent
Soil	*Maharashtra*	BIONCL P62	velutinous	Dark bluish green	Dark brown	Good	Present
Food	*Maharashtra*	BIONCL P63	Velutinous	Dark green	Dark brown	Moderate to good	present
Food	*Telangana state*	BIONCL P64	Floccose to velutinous	Creamy white	Yellow	Moderate to good	Present
Food	*Telangana state*	BIONCL P65	Floccose to velutinous	Bluish green	Pale yellow	Moderate to good	Absent
Soil	*Telangana state*	BIONCL P66	Velvety	Green	Pale yellow	Good	Present
Air	*Telangana state*	BIONCL P67	Floccose to velvety	Light green	Pale yellow	Moderate to good	Present
Air	*Telangana state*	BIONCL P68	Floccose	Creamy white	pale yellow	Weak to moderate	Absent
Air	*Telangana state*	BIONCL P69	Floccose	Creamy white	Pale yellow	Moderate to good	Absent

Food	*Telangana state*	BIONCL P70	Floccose	Green	Pale yellow	Moderate to good	Present
Soil	*Andra pradesh*	BIONCL P71	Floccose	Green	Pale yellow	Moderate to good	Present
Soil	*Andra pradesh*	BIONCL P72	Floccose	Green	Yellow	Moderate	Absent
Grain	*Andra pradesh*	BIONCL P73	Floccose	Green	Brownish yellow	Moderate to good	Present
Air	*Telangana state*	BIONCL P74	Floccose	Green	Pale yellow	Moderate to good	Absent
Soil	*Telangana state*	BIONCL P75	Velvety	Green	Pale yellow	Good	Present
Grain	*Maharashtra*	BIONCL P76	Velutinous	Dark green	Dark brown	Moderate to good	present
Soil	*Maharashtra*	BIONCL P77	Velutinous	Green	Brownish yellow	Moderate to good	Absent
Soil	*Maharashtra*	BIONCL P78	Floccose to velutinous	Green	Brownish yellow	Moderate to good	Absent
Soil	*Maharashtra*	BIONCL P79	Velutinous	Green	Pale yellow	Moderate to good	Present
Grain	*Maharashtra*	BIONCL P80	Floccose to velutinous	Light green	Pale yellow	Good	Absent
Soil	*Maharashtra*	BIONCL P81	Floccose	Whiteish green	Brown	Moderate to good	Absent
Soil	*Maharashtra*	BIONCL P82	Velutinous	Green	Brownish yellow	Moderate to good	Present
Soil	*Maharashtra*	BIONCL P83	Velutinous	Dark green	Brownish yellow	Moderate to good	Present
Soil	*Maharashtra*	BIONCL P84	Floccose	Dark green	Yellow	Good	Present
Grain	*Andra pradesh*	BIONCL P85	Velutinous	Green	Yellow	Moderate to good	Present
Food	*Gujarat*	BIONCL P86	Floccose to velutinous	Light green	Yellow	Moderate to good	Present
Food	*Telangana state*	BIONCL P87	Floccose	Green	Yellow	Good	Present
Food	*Maharashtra*	BIONCL P88	Floccose to velutinous	Green	Yellow	Good	Present
Food	*Maharashtra*	BIONCL P89	Floccose	Dark green	Pale yellow	Moderate to good	Present
Food	*Andra pradesh*	BIONCL P90	Velutinous	Light green	Pale yellow	Moderate to good	Present
Soil	*Andra pradesh*	BIONCL P91	Floccose	Dark green	Pale yellow	Moderate to good	Present
Soil	*Telangana state*	BIONCL P92	Velutinous	Dark green	Pale yellow	Good	Present
Soil	*Gujarat*	BIONCL P93	Velutinous	Dark green	Pale yellow	Moderate to good	Present
Soil	*Maharashtra*	BIONCL P94	Floccose to velutinous	Light green	Pale yellow	Moderate to good	Present

soil	*Andra pradesh*	BIONCL P95	Floccose	Whiteish green	Brown	Weak to moderate	Absent
Soil	*Andra pradesh*	BIONCL P96	Floccose	Whiteish green	Brownish yellow	Weak to moderate	Absent
Soil	*Telangana state*	BIONCL P97	Floccose to velutinous	Green	Brownish yellow	Weak to moderate	Absent
Soil	*Telangana state*	BIONCL P98	Floccose to velutinous	Whiteish green	Brownish yellow	Weak to moderate	Absent
Soil	*Maharasht ra*	BIONCL P99	Floccose to velutinous	Whiteish green	Brownish yellow	Moderate to good	Absent
Soil	*Andra pradesh*	BIONCL P100	Floccose to velutinous	Whiteish green	Brownish yellow	Weak to moderate	Absent
Soil	*Andra pradesh*	BIONCL P101	Floccose	Whiteish green	Brownish yellow	Weak to moderate	Absent
Soil	*Telangana state*	BIONCL P102	Floccose to velutinous	Whiteish green	Brownish yellow	Weak to moderate	Absent
Grain	*Maharasht ra*	BIONCL P103	Floccose to velutinous	Whiteish green	Brownish yellow	Moderate to good	Absent
Grain	*Gujarat*	BIONCL P104	Floccose	Green	Brownish yellow	Weak to moderate	Present
Grain	*Andra pradesh*	BIONCL P105	Floccose to velutinous	Whiteish green	Brownish yellow	Moderate to good	Present
Grain	*Andra pradesh*	BIONCL P106	Floccose	Whiteish green	Brownish yellow	Weak to moderate	Present
Soil	*Maharasht ra*	BIONCL P107	Floccose	Creamy white	Brownish yellow	Weak to moderate	Absent
Soil	*Maharasht ra*	BIONCL P108	Floccose to velutinous	White	Pale yellow	Good	Absent
Soil	*Gujarat*	BIONCL P109	Floccose	Green	Pale yellow	Good	Absent

Table S2. Antimicrobial test zone of inhibition

Group 1. Std PenV	Conc. (µg/mL)	Zone of inhibition (mm)
	1	19
	5	26
	10	30
	20	33
	30	35
	40	36
	50	37
**Group 2. Extracted PenV**	**Strain**	**Zone of inhibition (mm)**
	Std. PenV (50 µg/mL)	36
	BIONCL P49	27
	BIONCL P1	37
	BIONCL P60	27
	BIONCL P30	32
	BIONCL P18	37
	BIONCL P17	18
	BIONCL P31	32
	BIONCL P40	34
**Group 3. Cell free supernatant (CFS)**	**Strain**	**Zone of inhibition (mm)**
	Std. PenV (50 µg/mL)	36
	BIONCL P30	33
	BIONCL P49	30
	BIONCL P60	26
	BIONCL P40	35
	BIONCL P1	32

BIONCL P17	19
BIONCL P31	31
BIONCL P18	37

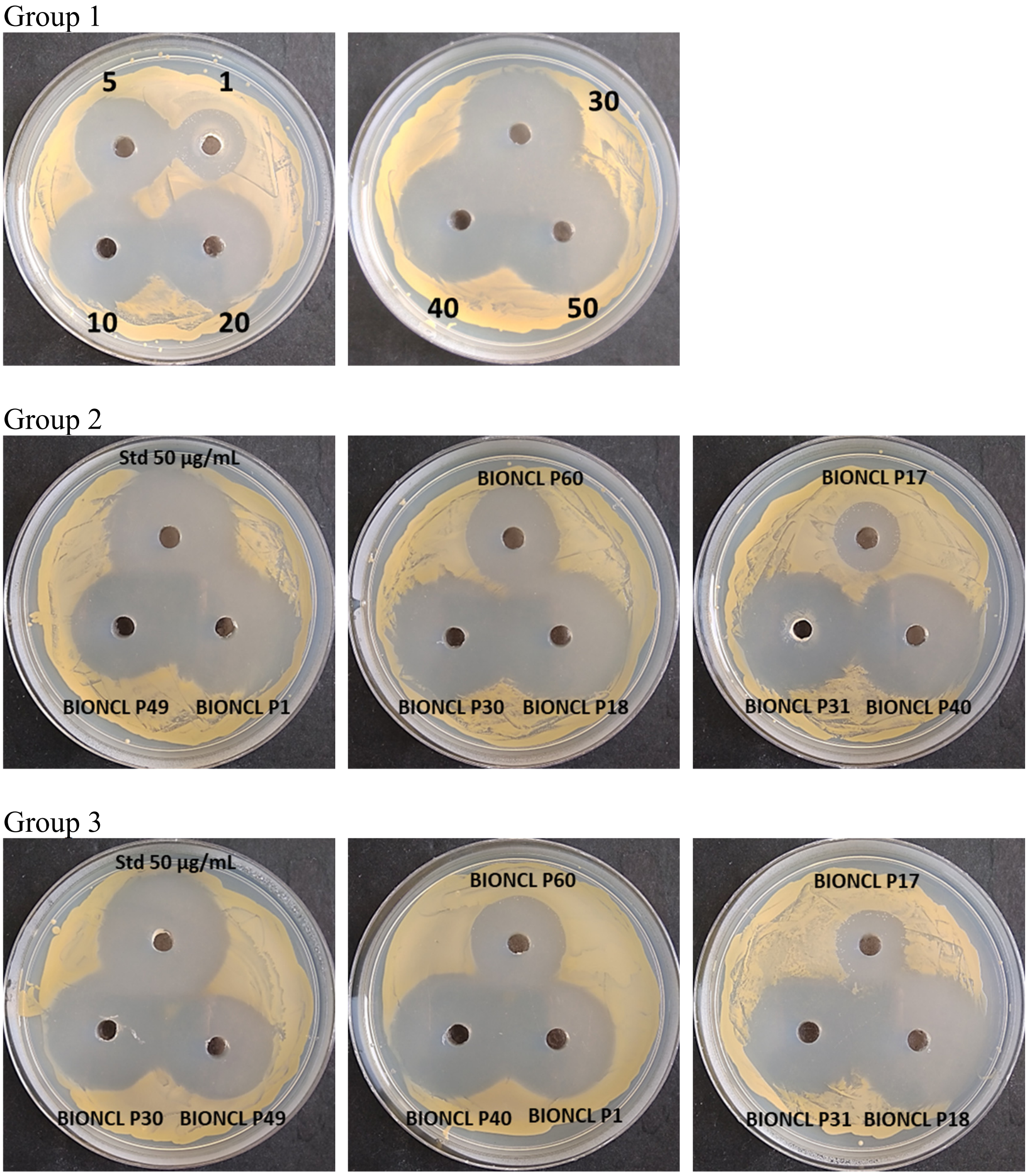

Figure S1. Antibiotic plate assay with *S. aureus* NCIM-2079 culture. Group 1- Std. PenV with varying concentration (1 µg/mL to 50 µg/mL), Group 2- Extracted PenV from culture broth, Group 3- Cell free supernatant (CFS).

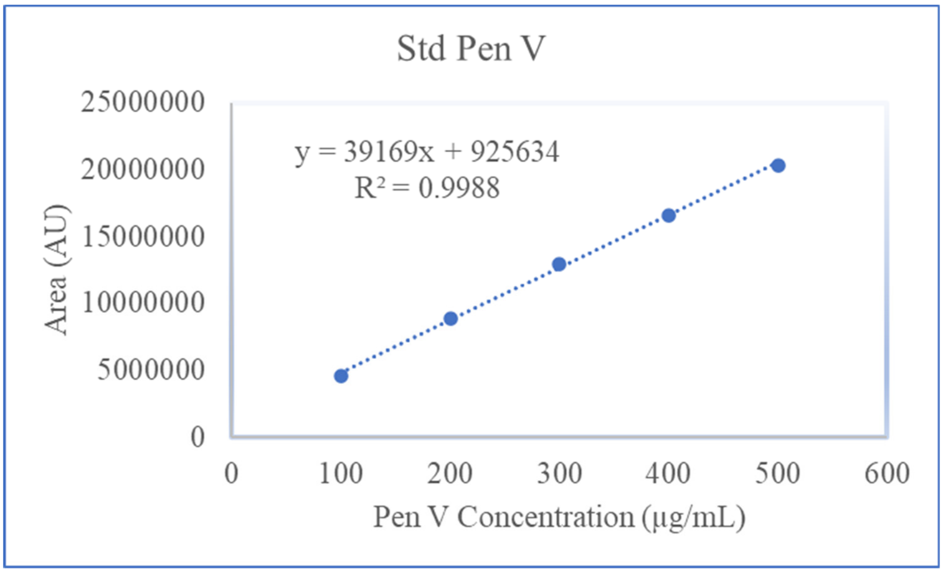
Std Pen V
y = 39169x + 925634
R² = 0.9988
Area (AU)
25000000
20000000
15000000
10000000
5000000
0
0
100
200
300
400
500
600
Pen V Concentration (µg/mL)

A

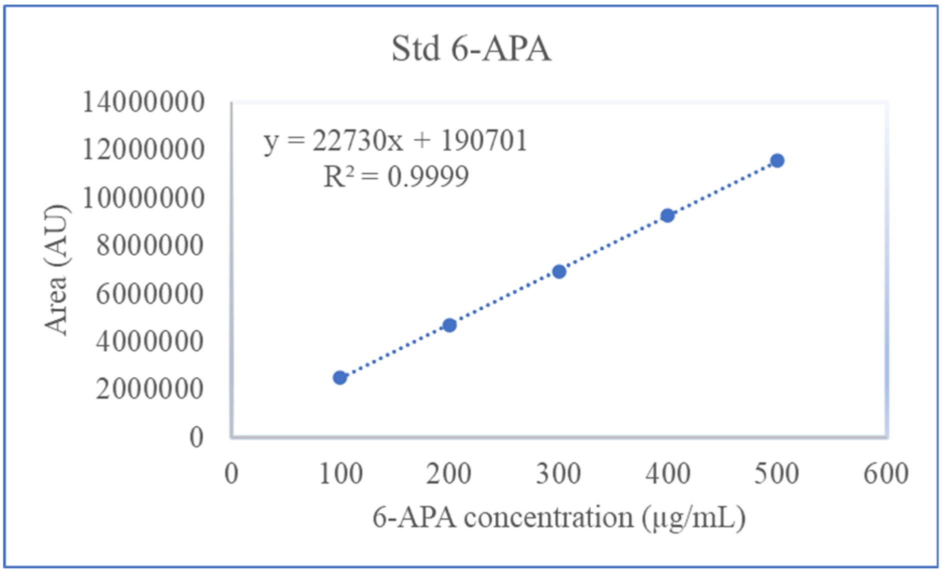
Std 6-APA
y = 22730x + 190701
R² = 0.9999
Area (AU)
14000000
12000000
10000000
8000000
6000000
4000000
2000000
0
0
100
200
300
400
500
600
6-APA concentration (µg/mL)

B

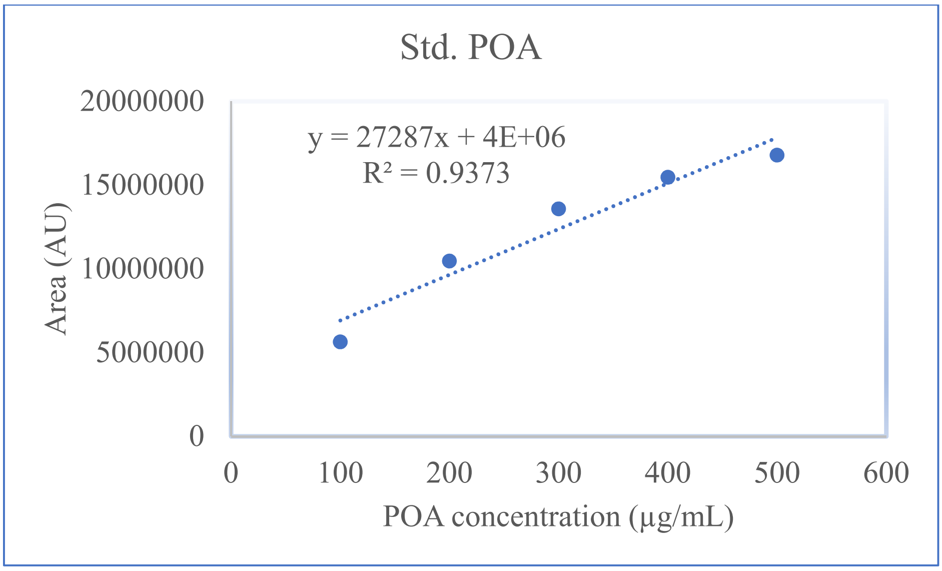

C

Figure S2. Calibration curve of A- Pen V, B- 6-APA, C- POA.
